# Exploring the Causal Relationship Between the Plasma Levels of MMP1 (Matrix Metalloproteinase‐1), MMP3, MMP7, MMP10, and MMP12 and Intervertebral Disc Degeneration: Mendelian Randomization

**DOI:** 10.1002/jsp2.70034

**Published:** 2025-01-07

**Authors:** Lin Sun, Kai‐Qing Yan, Qi Zhang, Ji Ma, Bo Shi, Xi‐Yuan Yang, Li‐Jun Li

**Affiliations:** ^1^ The Fifth Affiliated Hospital Shanxi Medical University Taiyuan People's Republic of China; ^2^ The Second Affiliated Hospital Shanxi Medical University Taiyuan People's Republic of China

**Keywords:** intervertebral disc degeneration, matrix metalloproteinase‐3, matrix metalloproteinases, Mendelian randomization

## Abstract

**Background:**

Several matrix metalloproteinases (MMPs) have been reported to be associated with intervertebral disc degeneration (IDD) in several previous studies. However, the causal relationship between MMPs and IDD remains unclear. In this study, Mendelian randomization (MR) was used to analyze the causal relationship between the plasma levels of multiple MMPs and the risk of IDD.

**Methods:**

The GWAS data of the plasma levels of MMP1, MMP3, MMP7, MMP10, and MMP12 were derived from the genome‐wide variation associations of 21 758 European individuals. The genetic associations of the variants with IDD were investigated in the largest genome‐wide association study from GWAS pipeline using Phesant derived variables from UKBiobank (1045 cases; 461 965 controls). We used a two‐sample MR method to evaluate the causal relationship between these five MMPs and IDD. The causal effects were examined by inverse variance weighted (IVW) test. And sensitivity analysis was performed using Q test of IVW and MR‐Egger, MR‐Egger‐intercept and MR‐PRESSO.

**Results:**

We found a significant correlation between increased the plasma level of MMP3 and an increased risk of IDD (IVW: OR 1.000564, 95% CI 1.0000304–1.00110; *p* = 0.0383). The heterogeneity test (MR‐Egger Q test: *p* = 0.346 and IVW Q test: *p* = 0.460) indicated that there was no heterogeneity in this instrumental variable on the surface. Also, no directional horizontal pleiotropy was observed in the MR analysis (MR‐Egger, *p* = 0.708 and MR‐PRESSO, *p* = 0.609). There was no significant correlation between the plasma levels of MMP1, MMP7, MMP10, and MMP12 and an increased risk of IDD.

**Conclusion:**

Our MR analysis found that there is a potential causal relationship between increased the plasma level of MMP3 and the risk of IDD in the European population. There is no potential causal relationship between the plasma levels of MMP1, MMP7, MMP10, and MMP12 and an increased risk of IDD.

## Graphical abstract

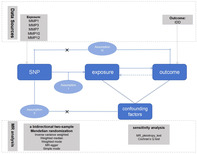

**Abbreviations**
CI: confidence interval
GWAS: genome‐wide association study
IDD: intervertebral disc degeneration
IVW: inverse‐variance weighted
MMP: matrix metalloproteinases
MR: Mendelian randomization
nSNP: number of single nucleotide polymorphism
OR: odds ratio
SE: standard error

## 1 Background

Low back pain (LBP) is a very common symptom. It occurs in various countries and all age groups and becomes the main cause of global disability [1]. Each year, the prevalence of LBP in the general adult population in the United States is 10%–30%, and the lifetime prevalence in American adults is as high as 65%–80% [2]. Globally, LBP is the leading global cause of years lived with disability. More attention is urgently needed to reduce this increasing burden and its impact on health and social systems [3]. It is estimated that healthcare expenditures in the United States for low back and neck pain, other musculoskeletal diseases, and diabetes account for the highest expenditures [4]. Lumbar IDD is one of the main causes of chronic LBP [5]. Magnetic resonance imaging‐related studies have found that an increased risk of all types of LBP is associated with all signs of IDD [6]. IDD can cause various signs and symptoms such as LBP, intervertebral disc herniation, and spinal stenosis, resulting in high social and economic costs. IDD is caused by many factors, including genetic factors, aging, mechanical injury, and malnutrition. The pathological changes of IDD mainly include the senescence and apoptosis of nucleus pulposus cells (NPCs), the progressive degeneration of extracellular matrix (ECM), the fibrosis of annulus fibrosus (AF), and inflammatory response. At present, IDD can be treated by conservative treatment and surgical treatment according to the patient's symptoms. However, these can only relieve pain and cannot reverse IDD and reconstruct the mechanical function of the spine. Therefore, early monitoring and possible biological treatment methods are particularly important [7]. MMPs are a family of zinc‐dependent neutral endopeptidases that can basically degrade all ECM components [8]. Previous studies have shown that the gene expression levels of MMP1, MMP3, MMP7, MMP10, and MMP12 will all have a certain degree of impact on IDD [9, 10, 11, 12, 13, 14, 15]. For example, MMP‐1 immunohistochemical scores in IDD tissues of patients after lumbar spine surgery show an independent correlation with histological degeneration scores in patients with cervical or lumbar IDD, indicating that MMP‐1 immunohistochemical scores can be used as an indicator of IVD degeneration [10]; In degenerated intervertebral disc tissue, MMP‐3 is significantly correlated with the histopathological changes of expressing intervertebral discs, and there is no correlation between MMP‐3 expression and age, gender, and pain duration. It is speculated that MMP‐3 may become a therapeutic target for IDD [11]; MMP‐7 induces MMP‐3 through mediation, and MMP‐3 is necessary for the production of macrophage chemokines and subsequent macrophage infiltration of the intervertebral disc leading to IDD [12]; MMP‐10 mRNA and IDD also show a significant correlation in cadaver samples [13]; The results of animal experiments on MMP12 show that it will affect the homeostasis of intervertebral disc cells and may lead to IDD [14]. Another related study shows that specific MMPs (MMP‐1, ‐3, ‐7, and ‐10) are upregulated in human degenerated IVD [15]. Due to the biases, confusions, and reverse causal relationships that are prone to occur in basic experiments, animal experiments, and cadaver studies, misleading results are often obtained. Therefore, it is of great significance to study the causal relationship between the gene expression levels of MMP1, MMP3, MMP7, MMP10, and MMP12 and IDD. Mendelian randomization (MR) is a statistical model that uses genetic variation as an instrumental variable, which can avoid potential confounding factors and reverse causal relationship biases brought by observational studies [16, 17, 18]. We selected MMP1, MMP3, MMP7, MMP10, and MMP12 due to the fact that relevant basic research and animal experimental studies have demonstrated a strong correlation between these MMPs and IDD [9, 10, 11, 12, 13, 14, 15]. Consequently, we hold the view that it is of crucial significance for investigating the causal relationship regarding the risk of IDD. Second, in terms of technology and data acquisition, there are already relatively mature detection methods for MMP1, MMP3, MMP7, MMP10, and MMP12. Currently, the genome‐wide association study (GWAS) data on the plasma levels of MMP1, MMP3, MMP7, MMP10, and MMP12 possess a large sample size. This facilitates our better conduct of MR studies and helps to reduce experimental errors and uncertainties. We conducted multiple two‐sample MR studies to analyze the causal relationship between the plasma levels of MMP1, MMP3, MMP7, MMP10, and MMP12 and the risk of IDD.

## 2 Methods

### 2.1 MR Assumptions

**FIGURE 1:**
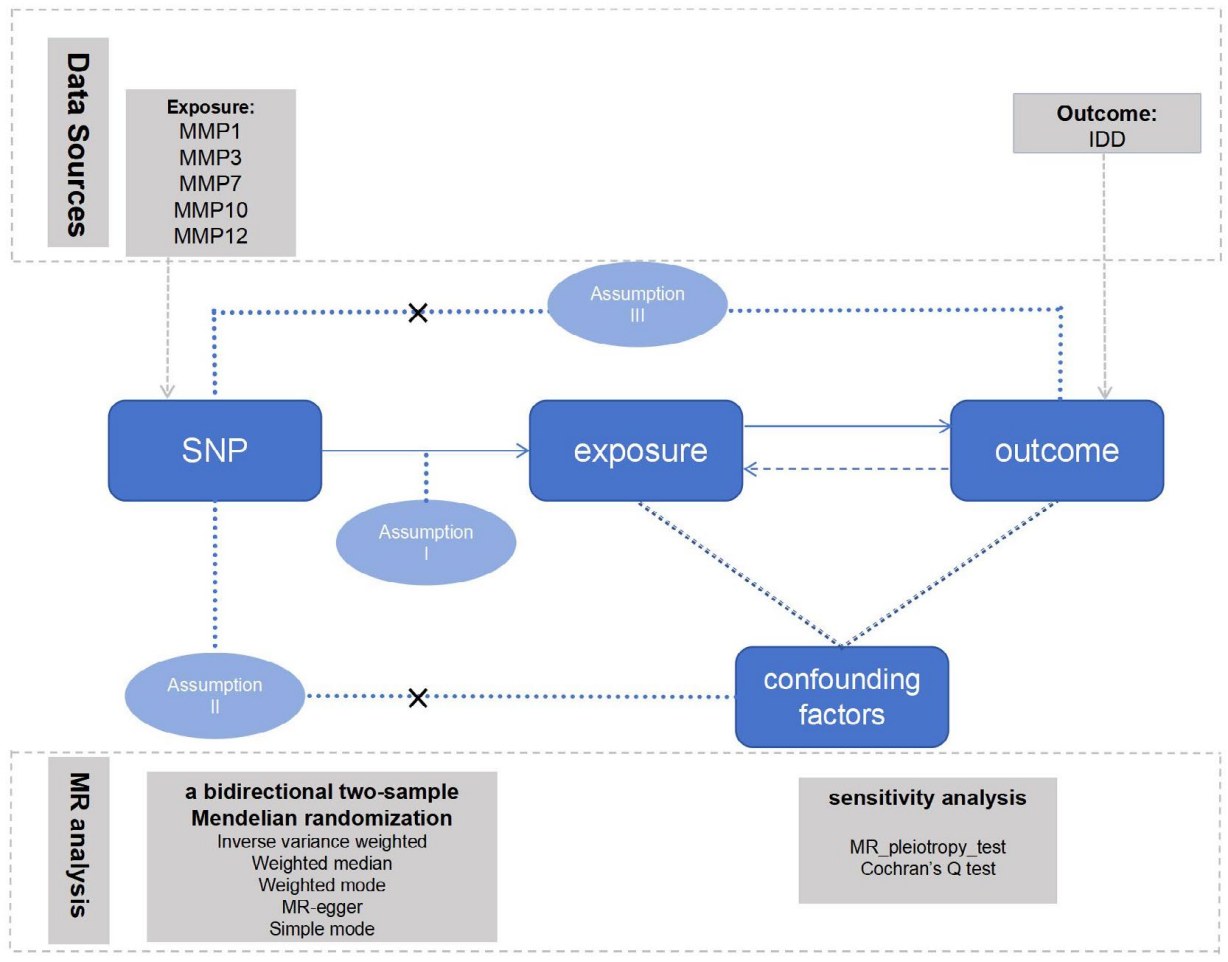
Three core assumptions of MR analysis in this study. IDD: intervertebral disc degeneration; IVW: inverse‐variance weighted; MMP1: matrix metalloproteinase‐1; MMP10: matrix metalloproteinase‐10; MMP12: matrix metalloproteinase‐12; MMP3: matrix metalloproteinase‐3; MMP7: matrix metalloproteinase‐7; MR: Mendelian randomization; SNP: single nucleotide polymorphism.

MR analysis has three key assumptions: (1) Association assumption: Genetic instrumental variables (IVs) are closely related to exposure factors; (2) Independence assumption: IVs are not associated with potential confounding factors; (3) Exclusivity assumption: IVs should affect outcome factors only through exposure factors. The research design is shown in Figure 1. In this study, two genome‐wide association study (GWAS) datasets were used to identify genetic significant single nucleotide polymorphisms (SNPs) for the plasma levels of MMP1, MMP3, MMP7, MMP10, and MMP12 and IDD [19].

### 2.2 GWAS Data Source

The genetic associations of IDD were investigated in the largest genome‐wide association study conducted in the GWAS pipeline using Phesant derived variables from UKBiobank (1045 cases; 461 965 controls). The diagnosis of IDD is based on the International Classification of Diseases ICD10: M51.3 is defined as thoracic, thoracolumbar, and lumbosacral IDD. The GWAS data of the plasma levels of MMP1, MMP3, MMP7, MMP10, and MMP12 are derived from the genome‐wide variation associations of these five MMPs in 21 758 European individuals.

### 2.3 Selection of Instrumental Variables

As genetic proxies for the plasma levels of MMP1, MMP3, MMP7, MMP10, and MMP12, SNPs with a genome‐wide significant threshold (*p* < 5 × 10^−8^) were used. However, when this threshold was applied, there were fewer than five genome‐wide significant SNPs in the GWAS results for all five MMPs. In this case, MR analysis would encounter issues such as low statistical power and weak instrumental variables. Therefore, a threshold of *p* < 5 × 10^−6^ was chosen to select SNPs and aggregate these SNPs. These SNPs were clustered together with a threshold of kb = 10 000 and *r* ^2^ = 0.001 to eliminate linkage disequilibrium, and the *F* statistics of each cytokine instrument were calculated. We set a threshold of *F* > 10. The software tool LDlink was utilized to examine the genomic region surrounding the instrumental variable, ensuring that there were no SNPs related to other potential confounding factors in a strong linkage disequilibrium state (the results of the LDlink examination can be found in the Supporting Information).

### 2.4 Data Analysis

The main analysis method of MR is inverse variance weighted (IVW) with random effects. In addition, MR‐Egger, weighted median, simple model, and weighted model were used as supplementary analysis methods. IVW is a meta‐method for analyzing the multi‐locus effects of multiple SNPs. It assumes that all SNPs are valid IVs and are completely independent of each other. For each SNP, IVW calculates its effect estimate on exposure and outcome, assigns weights to each SNP according to the variance of the effect estimate, and then obtains the overall causal effect estimate by weighted averaging. The *q* test of IVW and MR‐Egger was used to test the heterogeneity of the MR analysis results. *p* > 0.05 indicates no heterogeneity. MR‐Egger‐intercept was used to check for horizontal pleiotropy to ensure that genetic variations are independently associated with exposure and outcome. *p* > 0.05 indicates no pleiotropy. MR‐PRESSO was further applied to identify and exclude potential outliers. All MR analyses were performed using the “TwoSampleMR” and “Mendelian randomization” packages in R4.3.2 software.

### 2.5 Ethics Statement

The analytical studies received ethical approval from their respective local institutional review boards, and all participants provided written informed consent. The study was a secondary analysis of publicly available summary statistics and did not involve human subjects or personal information. As such, ethical approval was not required.

## 3 Results

**FIGURE 2:**
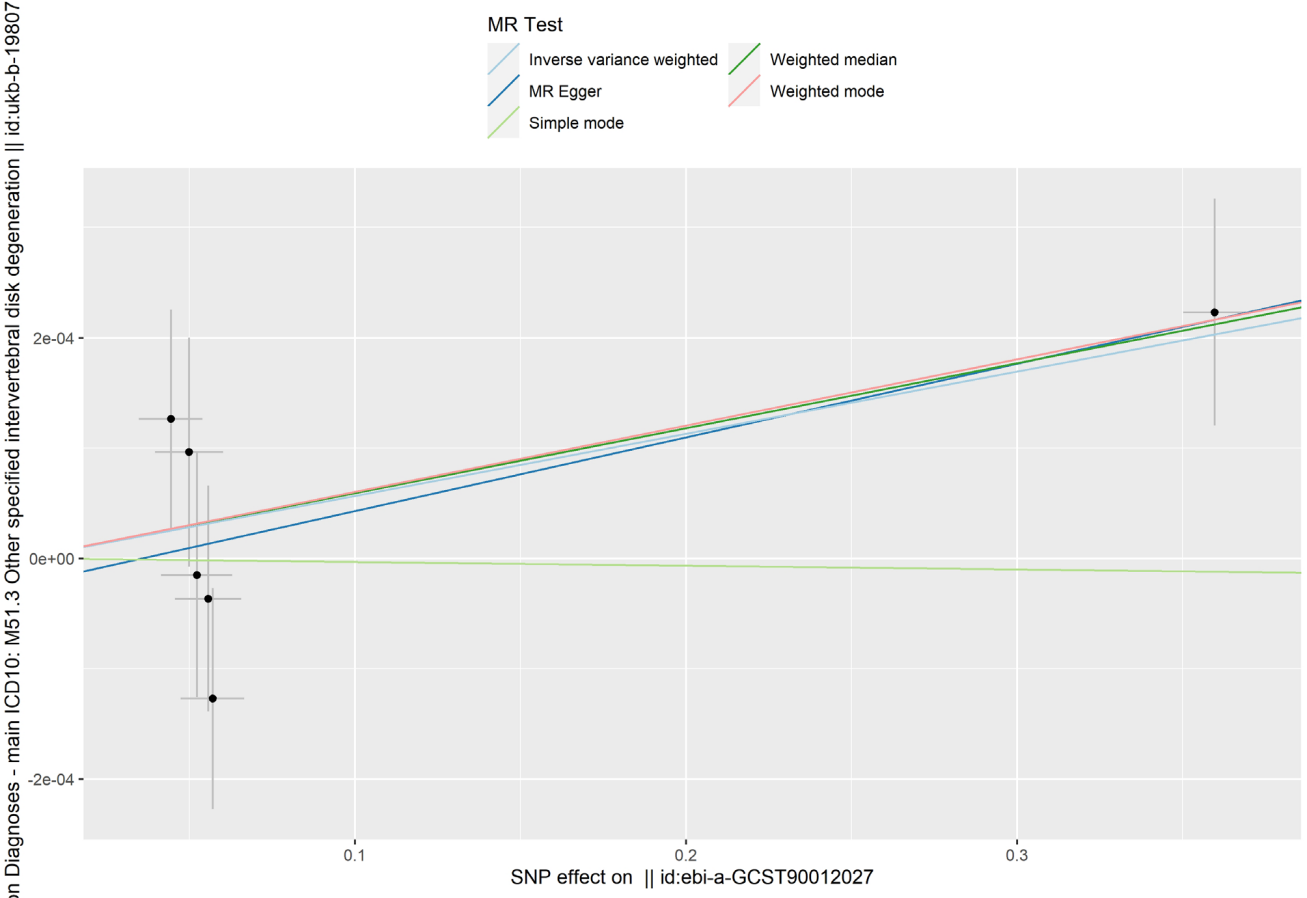
Scatter plot of genetic associations with MMP3 against the risk of IDD using different MR methods. IDD: intervertebral disc degeneration; IVW: inverse‐variance weighted; MMP3: matrix metalloproteinase‐3; MR: Mendelian randomization.

**FIGURE 3:**
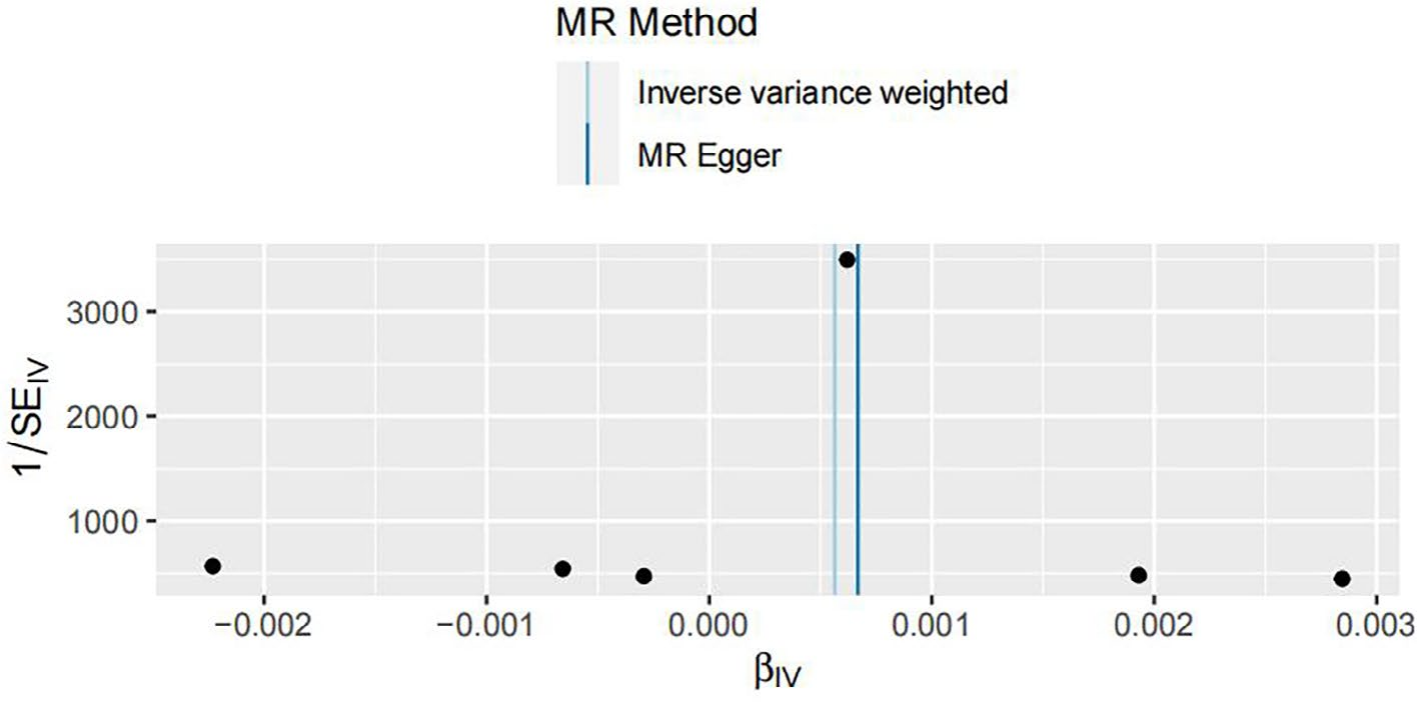
Plots of “funnel‐plot” analyses for MR analyses of the causal effect of MMP3 with the risk of IDD. IDD: intervertebral disc degeneration; IVW: inverse‐variance weighted; MMP3: matrix metalloproteinase‐3; MR: Mendelian randomization.

**FIGURE 4:**
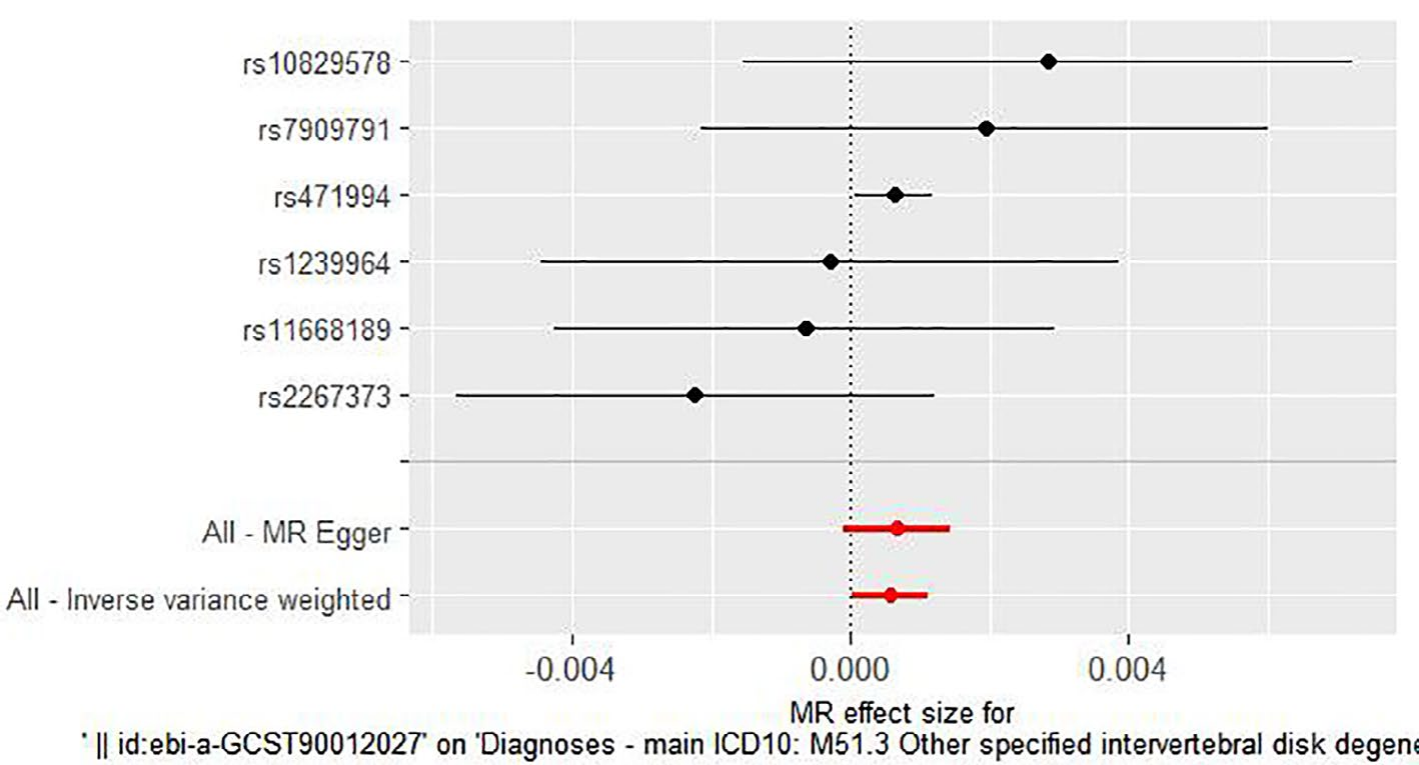
Plots of “forest‐plot” analyses for MR analyses of the causal effect of MMP3 with the risk of IDD. IDD: intervertebral disc degeneration; IVW: inverse‐variance weighted; MMP3: matrix metalloproteinase‐3; MR: Mendelian randomization.

IVW analysis showed a significant correlation between MMP3 among the five MMPs and an increased risk of IDD (IVW: OR 1.00056414906274, 95% CI 1.0000303921177–1.0010981908956; *p* = 0.0383) (Table 1). The heterogeneity test (MR‐Egger Q test: *p* = 0.346187915294615 and IVW Q test: *p* = 0.460021110026702) indicated that there was no heterogeneity in this instrumental variable on the surface. Also, no directional horizontal pleiotropy was observed in the MR analysis (MR‐Egger, *p* = 0.707963662 and MR‐PRESSO, *p* = 0.609). There was no significant correlation between the plasma levels of MMP1, MMP7, MMP10, and MMP12 and an increased risk of IDD (IVW: *p* = 0.39; *p* = 0.46; *p* = 0.87; *p* = 0.72) (Table 2) Please refer to the Supporting Information for details. These data can be found in the Supporting Information. The causal association of MMP3 with IDD is shown in Tables 1, 2, 3, 4 and Figures 2, 3, 4.

**TABLE 1:** MR estimates of the causal association between MMP3 and the risk of IDD.

Exposure	Outcome	Method	SE	*p*	OR	or_lci95	or_uci95
MMP3	IDD	MR‐Egger	3.87E‐04	0.159	1.00E+00	1.00E+00	1.00E+00
MMP3	IDD	Weighted median	2.75E‐04	0.032	1.00E+00	1.00E+00	1.00E+00
MMP3	IDD	IVW	2.72E‐04	0.038	1.00E+00	1.00E+00	1.00E+00
MMP3	IDD	Simple mode	9.07E‐04	0.972	1.00E+00	9.98E‐01	1.00E+00
MMP3	IDD	Weighted mode	3.04E‐04	0.105	1.00E+00	1.00E+00	1.00E+00

**TABLE 2:** IVW with five MMPs against the risk of IDD using MR method.

Exposure	Outcome	Method	*p*
MMP1	IDD	IVW	0.394
MMP3	IDD	IVW	0.038
MMP7	IDD	IVW	0.460
MMP10	IDD	IVW	0.88
MMP12	IDD	IVW	0.72

**TABLE 3:** Results of Cochran's Q test and MR‐Egger intercept.

id.exposure	id.outcome	Outcome	Exposure	Method	*Q*	Q_df	Q_pval
ebi‐a‐GCST90012027	ukb‐b‐19 807	IDD	MMP3	MR‐Egger	4.469	4	0.346
ebi‐a‐GCST90012027	ukb‐b‐19 807	IDD	MMP3	IVW	4.65	5	0.460

**TABLE 4:** Results of Egger_intercept.

id.exposure	id.outcome	Outcome	Exposure	egger_intercept	SE	*p*
ebi‐a‐GCST90012027	ukb‐b‐19 807	IDD	MMP3	−2.40E‐05	5.97E‐05	0.708

## 4 Discussion

IDD can cause various signs and symptoms such as LBP, IDD, and spinal stenosis, resulting in high social and economic costs [4]. So far, although the incidence of IDD is very high, our understanding of its etiology is not comprehensive enough and remains elusive [7]. In recent years, there is evidence that MMPs may be related to the progression of IDD, leading to the development of IDD [6, 9, 15]. This makes MMPs a key research direction. In this study, through two‐sample MR analysis, the potential association between MMPs and IDD was explored at the genetic level.

Our research results provide evidence for the causal relationship between increased the plasma level of MMP3 and an increased risk of IDD. In addition, there is no corresponding causal relationship between the plasma levels of MMP1, MMP7, MMP10, and MMP12 and an increased risk of IDD. First, speculation based on the relevance of biological mechanisms: Although currently only MMP‐3 is significantly associated with IDD, MMP‐1, MMP‐7, MMP‐10, and MMP‐12 all belong to the MMP family. From the perspective of biological functions, the members of this family have similar mechanisms of action in the process of ECM metabolism. For example, they may all be involved in the degradation process of the main components of the intervertebral disc, such as collagen and proteoglycans. Moreover, in the existing studies, the correlations between these specific MMPs and IDD have been found. Theoretically, they may have potential impacts on IDD through similar pathways or synergistic effects. Second, considering data integrity and exploratory research: Incorporating these MMPs into the analysis helps to provide a more comprehensive perspective for studying the relationship between the entire MMP family and IDD. In the initial stage of the research, we hope to construct a more complete network of potential risk factors through the analysis of multiple relevant MMPs. This can avoid omitting important associations. Even though some MMPs may not seem to have significant findings at present, with the deepening of the research or under different sample populations and experimental conditions, their potential connections with IDD may be discovered. In addition, from the perspective of data analysis: Incorporating multiple MMPs can serve as a means of comparison and verification. By observing the differences between them and MMP‐3 (which is known to be significantly associated with IDD) in the statistical model, it helps us to better understand the unique role of MMP‐3 therein, and can further explore whether there are any weak associations or regulatory relationships between other MMPs and IDD that have not yet been discovered. Finally, regarding the continuity of the research and future research directions: Considering the long‐term goals and serial nature of the research, the analysis of these MMPs in this study can lay the foundation for subsequent research. In the future, new biomarkers or intervention targets may be found to be related to these MMPs. Incorporating them into the analysis now can ensure the coherence of the research. For example, with the development of technology or the deepening of the understanding of disease mechanisms, these seemingly unrelated MMPs may show importance in gene–environment interactions, early diagnosis of the disease, or the exploration of new treatment strategies.

MR study represents a powerful instrument that is capable of facilitating the inference of causal relationships between exposure factors, such as MMP3 plasma level, and disease outcomes, like IDD. At present, MMPs are generally able to degrade all ECM components, which is known as ECM degradation [8]. It is well established that MicroRNA 15a (miR‐15a) targets and modulates genes implicated in cell proliferation and apoptosis. Some data suggest that exo‐miR‐15a promotes the chondrogenic differentiation of nucleus pulposus‐mesenchymal stem cells (NP‐MSCs) by downregulating MMP‐3 via the PI3K/Akt and Wnt3a/β‐catenin axes, thereby deferring the progression of IDD [20]. In the domain of drug research and development, if MMP3 is recognized as a crucial factor in IDD, inhibitors directed against MMP3 can be developed. By suppressing the activity of MMP3, the degradation of the ECM can be diminished, consequently delaying the process of IDD. Regarding screening strategies, for individuals with a family history of IDD or those engaged in high‐risk occupations (such as long‐term stooping or heavy lifting), the polymorphism of MMP3‐related genes or the MMP3 plasma level can be examined as an early screening marker. If a relatively high MMP3 plasma level or an elevated risk of related genes is detected, preemptive interventions can be implemented, such as modifying lifestyle and intensifying lumbar muscle exercises. In the aspect of early intervention, when an increase in MMP3 plasma level is detected but no overt IDD symptoms have emerged, physical therapy or pharmacological treatment can be employed to regulate the expression of MMP3. For instance, certain drugs with anti‐inflammatory and regulatory effects on ECM metabolism may prove effective. Simultaneously, rehabilitation training can be incorporated to enhance spinal stability and relieve the pressure on the intervertebral discs.

Some studies have shown that specific MMPs (MMP‐1, ‐3, ‐7, and ‐10) are upregulated in human degenerated IVD [15]. Some other related animal experiments also show that MMP12 may affect the homeostasis of intervertebral disc cells and may lead to IDD [14]. Although there is a connection between them, the causal relationship has always been unable to be clarified due to the limitations of previous research conditions. In the context of MR analysis:

First, the influence of gene pleiotropy: Different members of the MMP family may exhibit diverse gene pleiotropy. An association between MMP3 and IDD has been identified, whereas other MMPs show no significant differences. This could be attributed to the fact that although they share similarities in enzymatic functions (such as being involved in ECM metabolism), at the genetic level, their other physiological functions and signaling pathways vary. For instance, MMP3 might be precisely involved in the key signaling pathway or biological process related to IDD, while the processes in which other MMPs participate may have a relatively weak correlation with IDD. Second, the difference in expression regulation: The expression regulation mechanisms of MMPs are distinct. In intervertebral disc tissue, the expression of MMP3 may be highly regulated by specific transcription factors, microRNAs, or other regulatory molecules. These regulatory factors change during the process of IDD, resulting in alterations in MMP3 plasma level. In contrast, the expression of other MMPs is either not affected or less affected by these specific factors. For example, under certain cellular stress or inflammatory signals, the transcription of the MMP3 gene may be specifically activated, while the transcription of other MMP genes remains stable. Finally, the difference in tissue‐specific functions: Each MMP may possess different tissue‐specific functions in intervertebral disc tissue. Although they all can degrade ECM components, MMP3 may mainly act on the critical ECM components or cell types during the process of IDD. For example, MMP3 might preferentially target certain proteoglycans in the nucleus pulposus, while other MMPs may mainly function in other aspects such as collagen metabolism in the AF and have a relatively weak association with the main pathological process of IDD.

About genome‐wide significant SNPs of MMP3, First, we utilized The National Center for Biotechnology (NCBI), a public database in the United States, to conduct fine mapping on the involved SNPs, determining their specific locations on the genes, including whether they are in the coding region, promoter region, enhancer region, or intron region of the genes, and so on. It is worth noting that rs471994 is precisely located 8 kb near the MMP3 gene and may be at the position of an enhancer or a silencer. Moreover, in MR, the influence of this SNP on MMP3 is far greater than that of other SNPs. (See the Supporting Information for the NCBI inspection results.) Second, functional verification experiments can also be carried out. For SNP sites with the potential to regulate the expression of MMP3, functional verification can be performed in cell models or animal models. Additionally, multi‐omics data can be integrated to construct an association model of SNP—MMP3 expression—IDD. Finally, the verification of biological plausibility is conducted.

This study is the first to use the MR method to study the causal relationship between the plasma levels of MMP1, MMP3, MMP7, MMP10, and MMP12 and the risk of IDD. However, certain limitations should also be acknowledged. First, since the study individuals included in the GWAS data are of European descent, caution should be exercised when generalizing this conclusion to other ethnic groups. Second, there is a causal relationship between MMP3 and IDD, but there is a lack of relevant GWAS data to study the relationship between MMP3 variants and upstream and downstream factors and IDD. Finally, we set *p* < 5 × 10^−6^ as the threshold value for genome‐wide significance to select the IVs. This might introduce false‐positive variants, potentially biasing the results. Nonetheless, the *F* statistic of all selected IVs was more than 10, which reduced weak IV bias. However, MR is a statistical model that uses genetic variation as an instrumental variable, which can avoid potential confounding factors and reverse causal relationship biases brought by observational studies.

## 5 Conclusion

Our MR analysis found that there is a potential causal relationship between increased plasma level of MMP3 and the risk of IDD in the European population. There is no potential causal relationship between the plasma levels of MMP1, MMP7, MMP10, and MMP12 and an increased risk of IDD.

## Author Contributions

Lin Sun and Kai‐Qing Yan conceptualized and designed the study. Kai‐Qing Yan, Qi Zhang, Ji Ma, Bo Shi, Xi‐Yuan Yang, and Li‐Jun Li performed data analysis. Lin Sun wrote the manuscript. All authors contributed to the article and approved the submitted version.

## Conflicts of Interest

The authors declare no conflicts of interest.

## Supporting information


**Data S1.** Supporting Information.

**Data S2.** Supporting Information.

**Data S3.** Supporting Information.

## Data Availability

The datasets presented in this study can be found in online repositories. The names of repository/repositories and accession number(s) can be found in the article/Supporting Information.
