# Supplementary material for: Exploring the Causal Relationship Between the Plasma Levels of MMP1 (Matrix Metalloproteinase‐1), MMP3, MMP7, MMP10, and MMP12 and Intervertebral Disc Degeneration: Mendelian Randomization
Source: JOR Spine. 2025 Jan 7;8(1):e70034. doi: 10.1002/jsp2.70034 (PMC11705520; doi:10.1002/jsp2.70034)
Supplement: Supplementary file 2 — Data S2. Supporting Information. [file JSP2-8-e70034-s003.docx]

>lcl|NC_000011.10_cds_NP_002413.1_1 [gene=MMP3] [db_xref=CCDS:CCDS8323.1,Ensembl:ENSP00000299855.5,GeneID:4314] [protein=stromelysin-1 preproprotein] [protein_id=NP_002413.1] [location=complement(join(102836126..102836226,102837298..102837401,102838551..102838710,102839110..102839243,102840108..102840252,102840429..102840593,102842154..102842279,102842431..102842579,102842672..102842916,102843442..102843546))] [gbkey=CDS]

ATGAAGAGTCTTCCAATCCTACTGTTGCTGTGCGTGGCAGTTTGCTCAGCCTATCCATTGGATGGAGCTG

CAAGGGGTGAGGACACCAGCATGAACCTTGTTCAGAAATATCTAGAAAACTACTACGACCTCAAAAAAGA

TGTGAAACAGTTTGTTAGGAGAAAGGACAGTGGTCCTGTTGTTAAAAAAATCCGAGAAATGCAGAAGTTC

CTTGGATTGGAGGTGACGGGGAAGCTGGACTCCGACACTCTGGAGGTGATGCGCAAGCCCAGGTGTGGAG

TTCCTGATGTTGGTCACTTCAGAACCTTTCCTGGCATCCCGAAGTGGAGGAAAACCCACCTTACATACAG

GATTGTGAATTATACACCAGATTTGCCAAAAGATGCTGTTGATTCTGCTGTTGAGAAAGCTCTGAAAGTC

TGGGAAGAGGTGACTCCACTCACATTCTCCAGGCTGTATGAAGGAGAGGCTGATATAATGATCTCTTTTG

CAGTTAGAGAACATGGAGACTTTTACCCTTTTGATGGACCTGGAAATGTTTTGGCCCATGCCTATGCCCC

TGGGCCAGGGATTAATGGAGATGCCCACTTTGATGATGATGAACAATGGACAAAGGATACAACAGGGACC

AATTTATTTCTCGTTGCTGCTCATGAAATTGGCCACTCCCTGGGTCTCTTTCACTCAGCCAACACTGAAG

CTTTGATGTACCCACTCTATCACTCACTCACAGACCTGACTCGGTTCCGCCTGTCTCAAGATGATATAAA

TGGCATTCAGTCCCTCTATGGACCTCCCCCTGACTCCCCTGAGACCCCCCTGGTACCCACGGAACCTGTC

CCTCCAGAACCTGGGACGCCAGCCAACTGTGATCCTGCTTTGTCCTTTGATGCTGTCAGCACTCTGAGGG

GAGAAATCCTGATCTTTAAAGACAGGCACTTTTGGCGCAAATCCCTCAGGAAGCTTGAACCTGAATTGCA

TTTGATCTCTTCATTTTGGCCATCTCTTCCTTCAGGCGTGGATGCCGCATATGAAGTTACTAGCAAGGAC

CTCGTTTTCATTTTTAAAGGAAATCAATTCTGGGCTATCAGAGGAAATGAGGTACGAGCTGGATACCCAA

GAGGCATCCACACCCTAGGTTTCCCTCCAACCGTGAGGAAAATCGATGCAGCCATTTCTGATAAGGAAAA

GAACAAAACATATTTCTTTGTAGAGGACAAATACTGGAGATTTGATGAGAAGAGAAATTCCATGGAGCCA

GGCTTTCCCAAGCAAATAGCTGAAGACTTTCCAGGGATTGACTCAAAGATTGATGCTGTTTTTGAAGAAT

TTGGGTTCTTTTATTTCTTTACTGGATCTTCACAGTTGGAGTTTGACCCAAATGCAAAGAAAGTGACACA

CACTTTGAAGAGTAACAGCTGGCTTAATTGTTGA
