# Supplementary material for: Exploring the Causal Relationship Between the Plasma Levels of MMP1 (Matrix Metalloproteinase‐1), MMP3, MMP7, MMP10, and MMP12 and Intervertebral Disc Degeneration: Mendelian Randomization
Source: JOR Spine. 2025 Jan 7;8(1):e70034. doi: 10.1002/jsp2.70034 (PMC11705520; doi:10.1002/jsp2.70034)
Supplement: Supplementary file 3 — Data S3. Supporting Information. [file JSP2-8-e70034-s001.docx]

| rs10829578 | GRCh37.p13 chr 10 | NC_000010.10:g.130927071C>A |
| --- | --- | --- |
| rs7909791 | GRCh37.p13 chr 10 | NC_000010.10:g.105613178C>A |
| rs471994 | GRCh37.p13 chr 11 | NC_000011.9:g.102697731G>A |
| rs1239964 | GRCh37.p13 chr 18 | NC_000018.9:g.56928652A>C |
| rs11668189 | GRCh37.p13 chr 19 | NC_000019.9:g.54754103C>A |
| rs2267373 | GRCh37.p13 chr 22 | NC_000022.10:g.38600542C>A |
